# Loss of employment: when personal resources are not enough. The contribution of EMDR

**DOI:** 10.3402/ejpt.v4i0.22982

**Published:** 2013-10-30

## XIII ESTSS Conference: “Trauma and its clinical pathways: PTSD and beyond”, Bologna, June 2013 ORAL, JUNE 9 HALL LIZ

## Trauma e perdita

### Perdita del lavoro: quando le risorse personali non bastano. Il contributo dell'approccio EMDR   10.00–10.15

#### Elisa Faretta, Tiziana Agazzi and Valentina Zambon Centro Studi P.I.I.E.C., Milan, Italy

“La perdita del lavoro” è un tema di attualità importante in questo periodo di crisi economica soprattutto per l'impatto che tale evento stressante ha sulla sfera psicologica ed emotiva. Il licenziamento può essere vissuto come evento critico, “traumatico”, destabilizzante e potrebbe contribuire a sviluppare un senso di impotenza, frustrazione e di disadattamento fino all'esordio di un Disturbo dell'Adattamento o un Disturbo da Stress Post-Traumatico. Attraverso l'utilizzo dell'EMDR, trattamento evidence-based per il PTSD, si aiutano i pazienti a modificare le valutazioni cognitive negative di sé e a rielaborare le emozioni disfunzionali collegate agli eventi critici. Inoltre, seguendo il modello “passato-presente-futuro” si permette l'elaborazione dei ricordi collegati ai momenti più stressanti del licenziamento, delle situazioni nel presente che possono riattivare il trauma di quei momenti ed infine di preparare la persona ad affrontare le situazioni future in maniera più adattiva e positiva. Saranno riportati alcuni casi.

### Loss of employment: when personal resources are not enough. The contribution of EMDR

“The loss of employment” is a very important topical issue in this period of economic crisis, especially because of the impact that this stressful event has on psychological and emotional aspects. The dismissal could be considered as a critical and “traumatic” event that could destabilize and lead to the development of a sense of powerlessness, frustration, and maladjustment, and then to an adjustment disorder or PTSD. With EMDR, an evidence-based treatment for PTSD, we help patients change negative cognitions about themselves and reprocess dysfunctional emotions related to critical events such as job loss. By following the model of “past–present–future”, it is possible to promote the processing of memories connected to the most stressful moments, the situation in the present that can reactivate the trauma of those moments, and finally preparing them to face the future situations in a more adaptive and positive manner. Several cases will be reported.

